# Functionally Tailored Metal–Organic Framework Coatings for Mediating Ti Implant Osseointegration

**DOI:** 10.1002/advs.202303958

**Published:** 2023-09-13

**Authors:** Yuan Zhang, Zhuo Cheng, Zaiyang Liu, Xinkun Shen, Chunyuan Cai, Menghuan Li, Zhong Luo

**Affiliations:** ^1^ Joint Disease & Sport Medicine Centre Department of Orthopaedics Xinqiao Hospital Army Medical University Chongqing 400038 China; ^2^ School of Life Science Chongqing University Chongqing 400044 China; ^3^ Department of Orthopaedics Ruian People's Hospital The Third Affiliated Hospital of Wenzhou Medical University Wenzhou 325016 China

**Keywords:** bone‐implant interface, metal‐organic frameworks, osseointegration, titanium implants

## Abstract

Owing to their mechanical resilience and non‐toxicity, titanium implants are widely applied as the major treatment modality for the clinical intervention against bone fractures. However, the intrinsic bioinertness of Ti and its alloys often impedes the effective osseointegration of the implants, leading to severe adverse complications including implant loosening, detachment, and secondary bone damage. Consequently, new Ti implant engineering strategies are urgently needed to improve their osseointegration after implantation. Remarkably, metalorganic frameworks (MOFs) are a class of novel synthetic material consisting of coordinated metal species and organic ligands, which have demonstrated a plethora of favorable properties for modulating the interfacial properties of Ti implants. This review comprehensively summarizes the recent progress in the development of MOF‐coated Ti implants and highlights their potential utility for modulating the bio‐implant interface to improve implant osseointegration, of which the discussions are outlined according to their physical traits, chemical composition, and drug delivery capacity. A perspective is also provided in this review regarding the current limitations and future opportunities of MOF‐coated Ti implants for orthopedic applications. The insights in this review may facilitate the rational design of more advanced Ti implants with enhanced therapeutic performance and safety.

## Graphical abstract

This review provides a concise yet comprehensive summary on the recent progress in the development of metalorganic framework (MOF) coating for the modification of titanium‐based orthopedic implants. Pro‐osseointegration benefit of MOF coatings is discussed in‐depth according to their physical, biochemical, and drug delivery potential, which may facilitate the development of more advanced orthopedic implants with enhanced fracture‐healing capacity and safety.

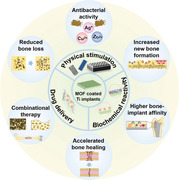

## 1 Introduction

Bone fracture is the most common injuries in daily life.^[^ ^1^ ^]^ Although some minor structural damage to the bone mass could be healed in a spontaneous manner on account of the regenerative property of bone tissues,^[^ ^2^ ^]^ there are many circumstances that require the implementation of orthopedic implants to support and stabilize the broken bones, which may prevent secondary bone damage and facilitate bone healing.^[^ ^3^ ^]^ Titanium (Ti) and its alloys are the most commonly used metallic materials for constructing bone implants. From an overall perspective, Ti‐based materials have high mechanical resilience, extreme resistance to chemical erosion and good biocompatibility, providing a promising solution to develop robust and durable implants for various indications. However, increasing evidence shows that the intrinsic bioinertness of Ti and its alloys may also profoundly impair their fracture fixation performance. Typically, the lack of pro‐osteogenic bioactivity in Ti‐based orthopedic implants would impede new bone formation at bone‐implant interface, which further leads to poor osseointegration and enhances the risk of implant loosening or displacement.^[^ ^4^ ^]^ The therapeutic performance of Ti‐based implants is further deteriorated in the context of osteodegenerative diseases such as osteoporosis or osteoarthritis. Specifically, although titanium implants are designed to permanently remain in human after implantation, clinical analysis has revealed that there are a series of adverse factors that may significantly shorten their service life. For instance, constant mechanical actions exerted on the Ti substrates would cause implant wear, leading to the release of debris and wear particles to cause allergic or cytotoxic reactions.^[^ ^5^ ^]^ Meanwhile, despite the bioinertness of Ti materials, they would still undergo gradually degradation in biological environment through galvanic corrosion to release Ti ions, which would significantly enhance the risk of peri‐implant inflammation, neurotoxicity and yellow nail syndrome.^[^ ^6^ ^]^ Furthermore, the insufficient bone formation at bone‐implant interface and implant‐induced destructive alterations in peri‐implant bone tissues would destabilize the implants in the implantation bed and cause loosening or detachment, which often require revision experiment or even surgical removal. From a general perspective, the service life of Ti implants is the collective result of implant design, tissue environment and biomechanical load. Typically, due to the frequent biomechanical movement and high susceptibility of pathogen invasion, Ti implants in oral regions usually have a functional lifespan of only ≈ 5–11 years.^[^ ^7^ ^]^ In contrast, the service life of total knee replacement implants is ≈ 15–20 years,^[^ ^8^ ^]^ while total hip replacement implants could last for ≈ 25 before revision surgery.^[^ ^9^ ^]^ Notably, due to the intense cyclic mechanical loading in human spines, the duration of spinal Ti implants is usually < 5 years.^[^ ^10^ ^]^ Consequently, new engineering strategies are urgently needed to improve the osseointegration of Ti‐based orthopedic implants to enhance their success rate under clinically relevant conditions, aiming to improving their osseointegration capacity while reducing the potential adverse effects.

Current insights collectively support the critical role of interfacial properties of orthopedic implants for mediating their osseointegration, which largely determine the recipient’ response to the implants as well as the behaviors of the implant in the implantation niche.^[^ ^11^ ^]^ Indeed, it is evident that the physical, structural and biochemical properties of the implant surface all have significant impact on their interaction with bone tissues and bone‐residing cell populations.^[^ ^12^ ^]^ For instance, it is reported that Ti implants with moderate surface hydrophilicity could substantially improve protein adhesion and be conductive to the binding of bone healing‐related cells for improving osseointegration, while superhydrophobic or superhydrophilic implant surfaces both impede the absorption of proteins and thus prevent effective osseointegration.^[^ ^13^ ^]^ Alternatively, implants with stiffer surfaces could more effectively promote the adhesion and expansion of bone‐related cells compared with their soft counterparts.^[^ ^14^ ^]^ Furthermore, recent studies collectively demonstrate that implants coated with bioactive molecules such as collagen, RGD peptide and dopamine on their surface could not only facilitate cell adhesion on implant surfaces but also potentially enable their osteogenic differentiation.^[^ ^15^ ^]^ Based on the principles above, various surface engineering strategies have been developed to optimize the interfacial properties of Ti‐based implants, aiming to enhance their pro‐osteogenic interaction with peri‐implant cells for accelerating osseointegration.

Metalorganic frameworks (MOFs) are a unique class of synthetic polymeric materials that are formed through the coordination between metal species and organic ligands.^[^ ^16^ ^]^ The repetitive extension of the coordination entities would lead to the formation of a vast coordination network with regular patterns. Owing to the intrinsic diversity in the applicable metal nodes and ligand struts, MOF materials readily present versatile 2D and 3D coordination configurations with programmable spatial geometry and chemical reactivities. Typically, due to the expanded coordination network, MOFs could demonstrate high porosity across atomic to micrometer scale.^[^ ^17^ ^]^ Meanwhile, the ligands could be modified with various functional groups to change their hydrophilicity/hydrophobicity.^[^ ^18^ ^]^ The rigidity of the ligand structs could also be altered to modulate the stiffness and mechanical strength of the MOF products.^[^ ^19^ ^]^ Taking advantage of these favorable features, MOFs have attracted broad interest for a myriad of applications including gas storage, catalysis, electronics, etc.^[^ ^20^ ^]^ Remarkably, MOFs have demonstrated a plethora of interesting properties including versatile selection of functional components, facile synthesis procedures, well‐defined 2D and 3D structures, scalable loading capacity and adjustable bioreactivity, which are highly favorable for various biomedical applications.^[^ ^21^ ^]^ For instance, there is already abundant evidence that the tunable porosity of MOF structures could be exploited as drug reservoirs for localized or systemic drug delivery.^[^ ^22^ ^]^ On the other hand, the introduction of metallic species could also endow novel bioreactivity that are rarely found in organic species, such as nanocatalytic capacity and photothermal potential.^[^ ^23^ ^]^ Interestingly, there is an increasing trend in the field of orthopedic research to deploy MOF‐based coatings on Ti implants as biofunctional interface, aiming to improve their bioactivity and facilitate osseointegration. Indeed, MOF‐based implant coatings have demonstrated several unique advantages for fracture repair, such as antibacterial activity, pro‐osteogenesis capacity, enhanced bone‐implant binding, etc. However, despite the burgeoning development in MOF‐modified Ti orthopedic implants, there is still no systematic review on this topic.

**Figure 1:**
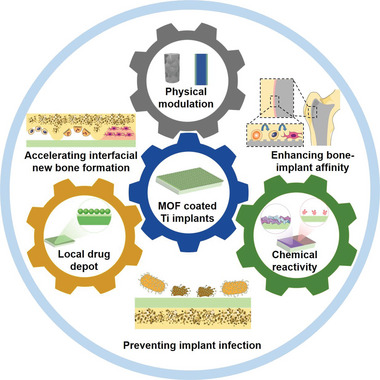
Schematic illustration of the pro‐osseointegration activities of functionally tailored MOF coating for Ti implant modification.

In this review, we will provide a comprehensive summary on the recent advances in the development of biofunctional MOF coatings for remodeling the bone‐implant interface (**Figure** **1**). Indeed, accumulating evidence collectively demonstrates that MOF‐based biomaterials have shown particular relevance for the functional engineering and optimization of Ti implant surface, of which the major merits include tunable surface morphology, tissue‐mimetic biomechanical profiles, metal/ligand‐dependent chemical reactivity, and controllable localized drug delivery capacity. By exploiting these clinically‐favorable structural, chemical, and biological features, MOF coating on Ti implants could readily convey an array of pro‐osseointegration advantages including enhanced bone‐implant adhesion strength, accelerated new bone formation at the bone‐implant interface and reduced risk of bacterial infection/biofilm formation, which may efficiently stabilize the implants after implantation and further facilitate fracture fixation and healing. A perspective is further included to discuss the challenges and potential opportunities in MOF‐coated Ti‐based orthopedic implants. It is anticipated that the insights in this study may facilitate the development of more advanced Ti‐based orthopedic implants with enhanced safety and therapeutic performance for fracture management in the clinics.

## 2 Bone Anatomy and Physiology

**Figure 2:**
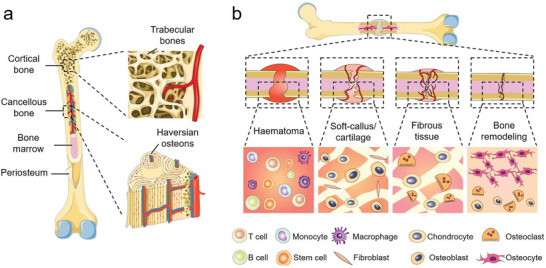
a) Schematic illustration of a typical long bone. b) Schematic demonstration of the major stages of natural bone healing process.

Bone is a highly mineralized connective tissue with many distinct properties and functions, which could be generally described as the hierarchical assembly of biominerals, cells and fibers.^[^ ^24^ ^]^ There are three major components in a bone, which include periosteum, osseous tissues and bone marrow, each with their unique structure, composition, and biological functions (**Figure** **2**a).^[^ ^25^ ^]^ The osseous tissues are some of the most important force bearing elements in human body, and fracture may occur when the applied forces or stresses exceed the bone strength.^[^ ^26^ ^]^ Notably, there are two types of osseous tissues, which are the cortical bones and trabecular bones. Fractures may occur in both types of osseous tissues.

Cortical bone is the dense and smooth outer surface of a bone with a low porosity < 10%, which forms a protective shell for encapsulating the bone marrow.^[^ ^27^ ^]^ The haversian systems in cortical bones are compactly arrayed and surrounded by lamellae in a concentric manner. Owing to the dense alignment of the haversian osteons, cortical bones tend to demonstrate high mechanical strength, which is crucial for enabling the biomechanical functions of the skeleton system including body framework, movement, and internal organ protection. Immense mechanical forces or stresses are usually required to cause breakage of cortical bones considering their mechanical resilience.^[^ ^28^ ^]^

On the other hand, cancellous bones fill the inner space of many types of bones, which present a highly porous structure with interconnected channel network.^[^ ^29^ ^]^ From a structural perspective, cancellous bones are characterized by a complex matrix of connected struts, which contains many irregular cavities filled with red bone marrow. Unlike the concentric arrangement of lamellae in cortical bones, lamellae in cancellous bones are parallelly aligned. Due to their sponge‐like structures, cancellous bones have much lower mechanical strength than cortical bones, but can still contribute to the overall rigidity and strength of the skeleton system. It is noteworthy that cancellous bones could be transformed to cortical bones through the osteogenic activity of osteoblasts. The cancellous bones account for ≈ 20% of the total osseous tissues in human body but are the most common sites for fractures to occur, such as the metaphyseal fractures or the vertebral compression fractures.^[^ ^30^ ^]^

Bone is the hardest tissue in human body and can withstand significant mechanical forces or stresses. However, various microscopic and macroscopic defects may occur when the mechanical load overwhelms the innate structural adaptation capacity of the bone tissues. Both cortical bones and cancellous bones have intrinsic self‐healing capability to repair minor damage in their nanocomposite structure.^[^ ^31^ ^]^ The natural bone healing process immediately starts following the fracture occurrence, which could be generally divided into several stages (Figure 2b). Bone breakage would damage the skeletal vasculature and cause local blood clotting, leading to the immediate formation of fracture haematomas that serve as contemporary templates for subsequent healing events.^[^ ^32^ ^]^ Meanwhile, the spontaneous release of tissue injury byproducts immediately initiates the inflammation response of ambient immune cells to recruit additional pro‐inflammatory cells and MSCs, thus orchestrating a bone‐regenerative microenvironment and forming fibrin‐rich granulation tissues.^[^ ^33^ ^]^ The recruited MSCs would further differentiate into pro‐osteogenic cells including fibroblasts, chondroblasts and osteoblasts, which is actuated by the stimulation of secreted signaling molecules such as bone morphogenetic proteins (BMPs).^[^ ^34^ ^]^ These cell populations would then act in a cooperative manner to regenerate bone tissues to connect the fracture ends. Specifically, fibroblasts could secrete abundant collagen to form reparative granuloma tissues between the fracture ends, which could act as the structural template for the subsequent ossification by osteoblasts and chondroblasts to form cancellous bones. Still, the newly formed immature bones would undergo continuous remodeling through the functional competition between osteoclasts and osteoblasts, until the normal bone structure is successfully restored.^[^ ^35^ ^]^

As described above, the healing and regeneration of damaged bone tissues is an intricate multi‐phase process, which is tightly regulated by multiple types of cells and signaling activities. However, it is also important to note that the self‐renewal capability of bone tissues is practically limited, and various types of additional treatment modalities are needed to stabilize and fix the implants in vivo, of which typical examples include bone cement and autologous/xenogeneic bone grafts.^[^ ^36^ ^]^ Nevertheless, these technologies have demonstrated several unsatisfactory issues during clinical practice. For instance, the application of bone cement or xenogeneic bone grafts may induce adverse immune reactions or allergy as well as production of wear debris, which may significantly enhance the risk of fixation failure and implant loosening. On the other hand, autologous bone grafts have intrinsically limited availability and are frequently associated with various forms of donor site morbidity. Moreover, they may present significantly different biomechanical properties compared to the original bones at the implantation site. Interestingly, functional implant coatings capable of activating the endogenous bone healing processes have become a promising solution to address these challenges. From a general perspective, these functional coatings could facilitate the recruitment of osteogenesis‐related cells to the bone‐implant interface and stimulate their osteoblastic activities by delivering specific physical, chemical, or biological cues, thus improving implant osseointegration in a regulated and non‐invasive manner. For instance, Liu et al. coated SrTiO_3_ (STO) implant surface with electropositive BiFeO_3_ (BFO) nanofilms, which could readily establish a built‐in electric field with the electronegative bone defect surface.^[^ ^37^ ^]^ The electric field between implant coating and bone tissues could significantly improve the adherence of proteins and MSCs as well as promoting their osteogenic differentiation, leading to accelerated implant osseointegration in mice and defect healing. Bai et al. coated Ti‐based implants with a composite peptide containing an osteogenic YGFGG sequence, which could reverse the polarization state of defect‐residing M1 macrophages into M2 phenotype to ameliorate the associated osteoclastic and osteolytic effects.^[^ ^38^ ^]^ These studies support the potential of rationally designed implant surface to improve osseointegration by exploiting endogenous bone healing mechanisms, suggesting their utility to achieve efficient and robust bone defect healing in the clinics.

## 3 MOF Coating for Ti Implant Functionalization

The implementation of MOF‐based biomaterials for tissue engineering has become an important topic with substantial clinical interest. Typically, MOFs are synthesized through the coordination‐driven assembly of atomic/nanoscale metal species with organic ligands, thus allowing the on‐demand tailoring of their physicochemical traits including pore width/volume, wettability, roughness and many other factors that may affect bio‐implant interaction.^[^ ^39^ ^]^ In addition, MOFs could be facilely synthesized by deploying bioreactive metal species as the coordination center including Zn, Ca, Cr, Fe, etc., further expanding their potential utility in bone‐related applications by endowing therapeutically favorable functions including cell recruitment, osteogenesis and anti‐infection.^[^ ^40^ ^]^ In this section, we will thoroughly discuss the major aspects of MOF‐based coating for improving the osseointegration of Ti implants, which is outlined according to their functional traits including: 1) optimizing the physical traits of bone‐implant interface, 2) drug depots for localized delivery of bioactive agents, and 3) MOF‐enabled biocatalytic reactions, aiming for boosting the endogenous bone formation processes while alleviating major risk factors that may cause implant loosening and detachment.

### 3.1 MOF‐Enabled Optimization of the Physical Traits of Bone‐Implant Interface

It is well established that bone tissues are highly sensitive to various physical cues including mechanical stress, interfacial topology, surface charge, etc., which have profound impact on the bone formation and remodeling processes.^[^ ^41^ ^]^ Considering the structural tailorability of MOF materials, there is considerable interest to modify Ti surface using synthetic MOF coating to alter their interfacial properties, aiming to facilitating vital pro‐healing events including protein/cell adhesion, osteoblastic differentiation/biomineralization and implant stabilization. From an overall perspective, the major approaches in this area could be classified as biomechanical, topological and physicochemical.

#### 3.1.1 MOF‐Based Biomechanical Modulation of Bone‐Implant Interface

**Figure 3:**
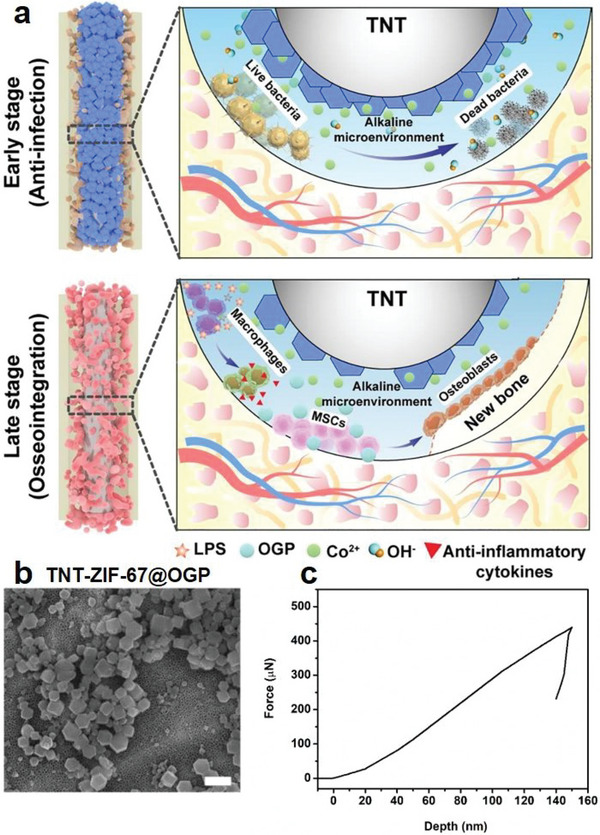
a) Schematic illustration of the pro‐osseointegration mechanism of the MOF‐coated TiO_2_ nanotube array. The ZIF‐67 MOF coating on TiO_2_ surface has similar elastic modulus to natural bone tissues and could thus avoid the stress shielding effect. Moreover, the MOF‐coating could be gradually degraded as the osseointegration progresses, thus avoiding bacterial infection in the early stages while enhancing the osteogenic activity of osteoblasts at later stages. b) SEM imaging showing the nanomorphology of the functionalized TiO_2_ nanotube array. c) Load‐displacement curve of TNT‐ZIF‐67@OGP, showing its similar mechanical elastic modulus with human femur. Reproduced with permission from Ref. [45]. Copyright 2021, Elsevier B.V.

The load transfer at the bone‐implant interface is an important factor affecting implant stability both in the short and long term.^[^ ^42^ ^]^ Specifically, Ti has much higher stiffness compared with natural bone tissues. On one hand, this is beneficial for stabilizing the broken bones that allows adequate bone rest and healing. On the other hand, the stiffness mismatch between Ti implants and bone tissues would cause load redistribution at the insertion site as the Ti components become the primary loading bearing unit, thus mechanically inducing significant bone atrophy and adverse bone remodeling. This effect is termed “stress shielding” and will induce significant bone loss and structural deterioration, eventually enhancing the risk of implant loosening and detachment.^[^ ^43^ ^]^ Interestingly, by rationally tuning the composition and synthetic conditions, the obtained MOFs could demonstrate bone‐like structure and elastic modulus, and it is thus anticipated that MOF‐coated bone implants could enable more uniform load distribution at the fracture site to avoid bone deterioration. Indeed, the clinical benefit of MOF‐enabled interfacial stiffness optimization of Ti implant is excellently demonstrated in a recent report by Wang et al., in which the authors sequentially wrapped 3D‐printed porous Ti6Al4V substrates first with biocompatible Mg‐containing MOF‐74 (Mg‐MOF‐74) and then with silk fibroin.^[^ ^44^ ^]^ Mechanical measurement results showed that the Mg‐MOF‐74 coating has an average elastic modulus and yield strength of ≈ 3.39 ± 0.04 GPa and 71.42 ± 2.47 MPa, Notably, the elastic modulus of the Mg‐MOF‐74 coating was significantly lower than human cortical bone tissues (5.44 ± 1.25 GPa), while its yield strength was also within the normal range for human cortical bones of ≈ 33–193 MPa. Consequently, the modification of Mg‐MOF‐74 layer onto porous Ti substrates could overcome the stiffness mismatch‐induced stress shielding effect frequently associated with common Ti‐based implants and facilitate new bone formation into the titanium structures, thus enhancing implant osseointegration for robust fracture fixation. In addition, the porous nature of Mg‐MOF‐74 also allows the facile incorporation of antiosteoporosis drug icariin, which could be released into the fracture site with Mg^2+^ ions in a silk fibroin‐controlled manner. Here icariin and Mg^2+^ could cooperatively induce M0‐M2 macrophage polarization through inhibiting notch signaling and thus abolish excessive bone resorption. Based on the merits above, the MOF‐coated Ti implants showed superior osseointegration efficiency compared with pristine porous Ti samples even under osteoporotic conditions. Similar benefit was also demonstrated in the study by Tao et al., in which the authors synthesized osteogenic growth peptide (OGP)‐integrated cobalt‐doped zeolitic imidazolate frameworks‐67 (ZIF‐67) coating for the modification of TiO_2_ nanotubes (**Figure** **3**).^[^ ^45^ ^]^ The authors first comparatively analyzed the elastic modulus of the ZIF‐67 coating to human femur to determine its capacity to circumvent stress shielding effect after implantation into femur defects. Specifically, results of the nanoindentation test based on load‐displacement analysis showed that the biofunctional ZIF‐67 coating presented an average elastic modulus of ≈ 16 GPa, which was evidently lower than the tensile elastic modulus of human femur of ≈ 18 GPa and could thus avoid the mechanically induced bone loss and deterioration. Furthermore, the biofunctional MOF coating could be gradually degraded in the acidic fracture bone microenvironment to release Co^2+^ ions and OGP. Here the Co^2+^ ions could demonstrate broad spectrum antibacterial property to prevent postoperative infection or biofilm formation, while the concurrently released OGP could induce the osteoblastic differentiation of ambient MSCs to promote their osteogenic potential. In addition, the acidity‐induced MOF hydrolysis would also scavenge excessive protons in the bone microenvironment and cause in‐situ pH reversal, which would reprogram the local pro‐inflammatory M1 macrophages into anti‐inflammatory M2 phenotype to alleviate the inflammation‐associated bone damage. These effects could act in a synergistic manner to facilitate robust osseointegration of implanted TiO_2_ nanotubes. Over all, these insights collectively supported the feasibility of employing tailored MOF coating to regulate the surface elastic modulus of Ti implants to facilitate osseointegration.

#### 3.1.2 MOF‐Based Topological Modulation of Bone‐Implant Interface

Alternative to the impact on the distribution of mechanical load, the micro‐topological features of implant surface could also affect osseointegration by mediating various mechanosignaling pathways, thus imposing regulatory effect on various osteogenesis events including adhesion, differentiation and biomineralization of bone cells.^[^ ^46^ ^]^ The most commonly explored micro‐topological features of Ti implants are their surface roughness and porosity. Specifically, abundant evidence demonstrates that high surface roughness would substantially increase the contact area between bone tissues and implants, thus elevating the total association forces in between. Meanwhile, elevating the implant surface roughness would also increase the surface energy to facilitate the adhesion of various serum or cell‐bound proteins. These merits are beneficial for improving cell migration and adhesion to implant surface.^[^ ^47^ ^]^ In addition, recent insights reveal that high surface roughness would induce the adaptive remodeling of the cytoskeleton of adhered cells to activate the downstream mechanotransduction pathways such as YAP‐TAZ and RhoA‐ROCK‐MLCK pathways, further stimulating the expression of osteogenesis‐related proteins to accelerate osseointegration.^[^ ^48^ ^]^ Interestingly, the tailorable structure and morphology of functional MOF coatings provides facile approaches to modulate the micro‐morphological properties of implant surface without tedious metal engineering processes. Typically, Dehnavi et al. immobilized chitosan nanofibers onto Ti implant surface to provide a highly porous substrate layer for anchoring silver‐based MOFs.^[^ ^49^ ^]^ The composite coating presented tissue‐like channeled network to potentiate nutrient supply as well as rapid waste exchange. Notably, the functionalization of the silver‐MOF‐based coating induced a > 700% increase in the surface implant roughness compared with bare Ti samples according to atomic force microscopy, which significantly improved the attachment of osteoblasts as well as enhancing their osteogenic potential without inducing obvious cytotoxicity. Moreover, the MOF‐bound Ag^+^ ions could exert potent broad‐spectrum antibacterial activity to inhibit the growth of both Gram‐positive and Gram‐negative bacteria. Similarly, Si et al. developed a simple and low‐cost strategy to synthesize MOF‐based multifunctional coatings for Ti implant modification.^[^ ^50^ ^]^ The authors reported that the controlled pyrolysis of ZIF‐8 and Cu precursors could lead to in situ generation of CuO@ZnO nanocomposites in ZIF‐8 MOF structures, which were further adhered onto polydopamine‐modified Ti surface. The MOF‐coating onto PDA‐modified Ti surface induced a dramatic increase in the surface roughness value from 1.97 nm in pristine Ti to 52.3 nm in MOF‐coated Ti surface. The elevated surface roughness in MOF‐coated Ti implants substantially contributed to the recruitment of human bone marrow mesenchymal stem cells (hBMSCs) to the implant surface and boosted their osteogenic potential by promoting their osteoblastic differentiation and activating the osteogenesis‐related genes. It is also important to note that due to the pyrolysis‐induced formation of CuO@ZnO nanocomposites, the MOF‐based coating showed enhanced in vivo stability compared to pristine Cu‐doped ZIF‐8 due to the substantially reduce ion leakage rate, which is beneficial for enabling long‐term pro‐osseointegration function and sustained antibacterial activity. Insights from these studies above immediately suggest that MOF functionalization could be employed to enhance the surface roughness of Ti implants to endow good osteoinductive properties while also introducing other novel pro‐osseointegration functions.

**Figure 4:**
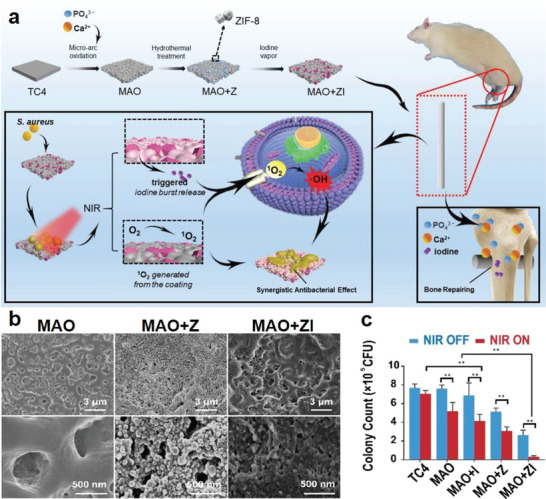
a) Porous MOF coating on MAO‐treated Ti surface substantially improved cell/protein adhesion to facilitate the subsequent osteogenic activity. Moreover, the multifunctional MOF coating allows photodynamic activity and iodine release under NIR treatment, leading to synergistic antibacterial effect. b) SEM imaging results on the surface morphology of the MAO‐treated Ti substrates. c) Photodynamic antibacterial activity of the MOF‐coated Ti implants. Reproduced with permission from Ref. [52]. Copyright 2021, Wiley‐VCH.

Alternatively, it is suggested that cells are prone to adhere to surfaces with higher porosity due to the increasing surface areas. There are also reports that increasing surface porosity of implants may enhance local vascularization as well as facilitate the nutrient supply and waste exchange of adhered cells, both of which are inducive to robust osseointegration of Ti implants.^[^ ^51^ ^]^ However, increasing the porosity of Ti implants would inevitably reduce their mechanical strength and enhance the risk of implant breakage or deformation, thus increasing the risk of secondary damage to the patients. Interestingly, the introduction of MOF coating onto Ti implants provides a facile approach to solve this dilemma. The intrinsic porosity of MOF components allowed the construction of porous structures on Ti surface with controlled pore volumes and widths. Moreover, the surface attachment of MOF coating would not affect the mechanical resilience of the Ti substrate. Typically, Teng et al. successfully immobilized porous ZIF‐8 MOFs on micro arc oxidized Ti surface via hydrothermal strategy and further doped iodine into MOF structures via vapor deposition to expand their clinical utility (**Figure** **4**).^[^ ^52^ ^]^ The micro arc oxidation pretreatment could significantly enhance the complexation between MOF coating and Ti substrates to enable stable MOF binding. SEM imaging immediately revealed the formation of well‐defined porous structures on titanium surface, which potently supported the migration and osteogenic differentiation of bone marrow stromal cells both in vitro and in vivo to stimulate new bone formation at the bone‐implant interface, leading to robust osteointegration after implantation. Moreover, ZIF‐8 MOF could demonstrate potent photodynamic activity under NIR illumination at 808 nm to kill local bacteria, which could synergize with the antibacterial activity of iodine to effectively prevent local infection. The pro‐osseointegration effect of porous MOF coating on Ti implants was also consistently supported in the study by Zhang et al., in which the authors anchored ZIF‐8 nanocrystals onto the surface of akali‐heat‐treated Ti implants in a quantitative manner and studied their impact on implant performance.^[^ ^53^ ^]^ The in vitro and in vivo characterization results demonstrated that the ZIF‐8 MOF coating conveyed a series of clinically favorable benefits, such enhanced expression of osteogenic genes including Alp, Col1, Opg, and Runx2 as well as enhanced secretion of osteogenesis‐related proteins including ALP and OPG in preosteoblast cells, increasing collagen deposition and accelerated osteoblastic biomineralization, eventually leading to substantially improved implant osseointegration compared with pure Ti samples.

#### 3.1.3 MOF‐Based Physicochemical Modulation of Bone‐Ti Interface

The physicochemical properties of Ti implant surface are crucial for determining their interaction with various biomacromolecules and cells, thus imposing substantial impact on the recruitment of osteogenesis‐related cell populations to the bone‐implant interface as well as their subsequent proliferation, differentiation and biomineralization.^[^ ^54^ ^]^ The most extensively studied physicochemical properties of Ti implant surfaces including: 1) surface hydrophilicity and 2) surface charge status. Indeed, water absorption occurs immediately following the implantation of the Ti implants that leads to the hydration of the implant surface. These surface‐bound water molecules present crucial roles mediating the interaction between Ti surface and ambient proteins, and it is commonly accepted that low surface hydrophilicity would impede protein/cell absorption and impair the subsequent osseointegration efficacy. This is also a major contributing factor to Ti implant failure in the clinics as pure Ti is highly hydrophobic with low surface wettability, while there is increasing evidence that enhancing the surface hydrophilicity could facilitate protein/cell adhesion and improve osseointegration.^[^ ^55^ ^]^ Nevertheless, there are also reports that excessive surface hydrophilicity may impair the osseointegration efficacy as it weakens the binding strength between implant surface and adhered cells.^[^ ^56^ ^]^ Therefore, it is important to maintain the surface hydrophilicity of Ti implants within a certain range to achieve optimal pro‐osteogenesis performance, although the exact details are still not clear and need to be investigated on a case‐by‐case basis. Typically, Wu et al. in situ generated bio‐derived MOFs based on Zn^2+^ ions and adenine columns on akali‐heat‐treated microroughened Ti surface to improve its osseointegration performance. The bioMOF modification induced a significant decrease in the surface contact angle values from the 119.2° in pure Ti to 22.9° in bioMOF‐coated Ti implants.^[^ ^57^ ^]^ Consequently, the bioMOF modification substantially improved the adhesion and osteogenic activity of osteoblasts on implant surface as well as enhanced the overall biocompatibility in vitro and in vivo. Furthermore, the bioMOF coating could be gradually degraded in vivo to release Zn^2+^ for stimulating osteogenesis‐related gene expression. Zhang et al. mimicked the non‐iridescent coloration mechanism in male eastern bluebird and developed supramolecular MOF structures with synergistic antibacterial and osteogenic capacities for TI implant modification.^[^ ^58^ ^]^ The multiscale supramolecular structure was constructed through the Lewis acidbase adduct formation between Au25(6‐mercaptohexanoic acid)18 nanoclusters and phytic acid (PA)‐metal complexes, which could reduce the surface contact angle of Ti surface from 91.4° in untreated Ti to 28.2° in MOF‐coated Ti surface, thus facilitating the recruitment and osteogenic differentiation of preosteoblastic cells. Notably, the abundant phosphate groups in phytic acid contents could further provide numerous chelation sites for ambient Ca^2+^ ions to accelerate biomineralization of recruited cells, while the coordinated metal ions such as Cu^2+^ and Zn^2+^ could efficiently kill local bacteria to prevent biofilm formation. Owing to the merits above, the MOF‐coated Ti implants showed significantly higher pro‐osteogenic activity with negligible infection‐induced inflammation in vivo.

**Figure 5:**
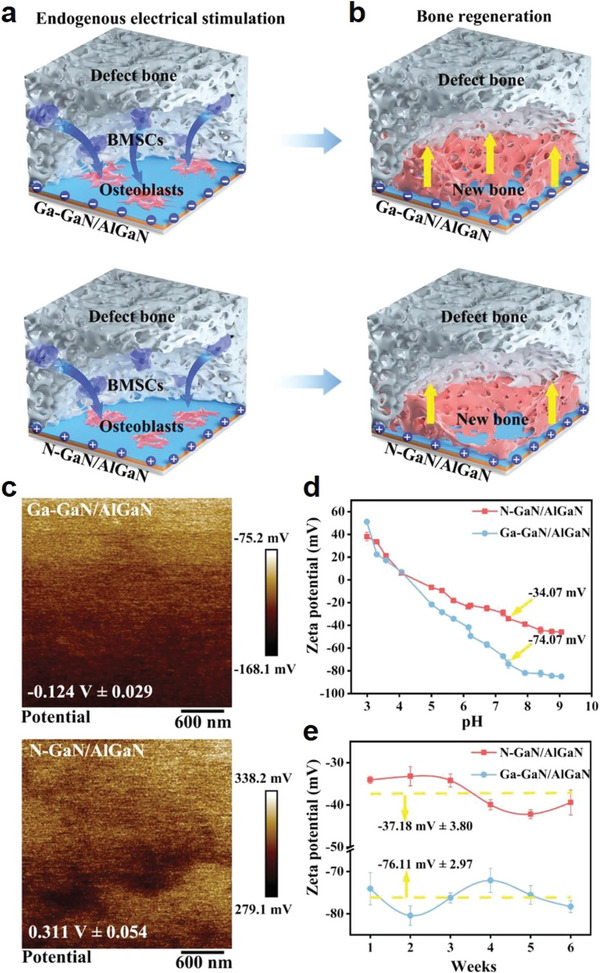
a,b) Mechanism of the GaN/AlGaN MOF‐coating mediated electrical stimulation for enhanced osseointegration. The negatively charged implant surface could attract MSCs and promote their differentiation to osteoblasts to promote new bone formation at bone‐implant interface. c) Surface potential of Ga‐GaN/AlGaN and N‐GaN/AlGaN coatings. d) Zeta potential values of Ga‐GaN/AlGaN and N‐GaN/AlGaN coatings. e) Stability of Ga‐GaN/AlGaN and N‐GaN/AlGaN coatings in DMEM for up to 6 weeks according to zeta potential analysis. Reproduced with permission from Ref. [59]. Copyright 2021, Wiley‐VCH.

Water is a strong dielectric medium and the interaction between water molecules and Ti implant surface would induce charge storage at the bone‐implant interface, which has attracted significant interest for profiling the potential impact on the recruitment of osteogenesis‐related cells and their osteogenic functions on implant surface. However, current investigations on the pro‐osseointegration function of interfacial charge storage is still not conclusive. Typically, Zhang et al. designed Ga‐polarity and N‐polarity GaN/AlGaN nanolayers with opposite polarity for the sequential modification of inorganic substrates and found that the GaN/AlGaN coating with the greatest negative surface potential of ⁻74.07 mV at pH 7.4 induced the highest stimulatory effect on the recruitment and osteogenic differentiation of bone mesenchymal stem cells (**Figure** **5**).^[^ ^59^ ^]^ Specifically, the negatively‐charged coating could attract ambient MSCs via galvanotaxis effects and enhanced their osteogenic function by activating multiple osteogenesis‐related pathways including focal adhesion, ECM‐receptor interaction, TGFβ signaling and PI3K‐AKT signaling, leading to accelerated bone defect healing after implantation. In contrast, Liu et al. reported that the charge status of bone defect walls was mostly negative, and the construction of electropositive ferroelectric BiFeO_3_ on SrTiO_3_ surfaces could establish a built‐in electrical field to both accelerate the bone formation rate at the defect site and improve the quality of the newly formed bone, possibly through the activation of PI3K‐AKT axis in recruited MSCs.^[^ ^37^ ^]^ These studies immediately suggested the complexity of electric signal‐regulated cell behaviors in the bone microenvironment, and it is anticipated that further studies in this area may significantly boost the pharmacological development toward enhanced fracture treatment.

### 3.2 Therapeutic Activities of Metal Species in MOF Coatings on Ti Implant Surface

It is well established that MOF structures are synthesized through the controlled and spontaneous coordination between organic ligands and metal ions/nanoclusters. Interestingly, the metal species in MOF structures could offer intrinsic biochemical reactivity that are not commonly found in organic materials, which provide unprecedented opportunities for introducing novel pro‐osseointegration functionalities into Ti implants.^[^ ^60^ ^]^ From an overall perspective, the primary therapeutic function of metal components in MOF coatings include (1) stimulating osteogenic functions of osteogenesis‐related cells and (2) inhibiting local biofilm formation and infection after implantation.

#### 3.2.1 MOF Coatings as Sources of Osteogenesis‐Stimulatory Metal Ions for Stimulating Peri‐Implant Osteogenesis

**Figure 6:**
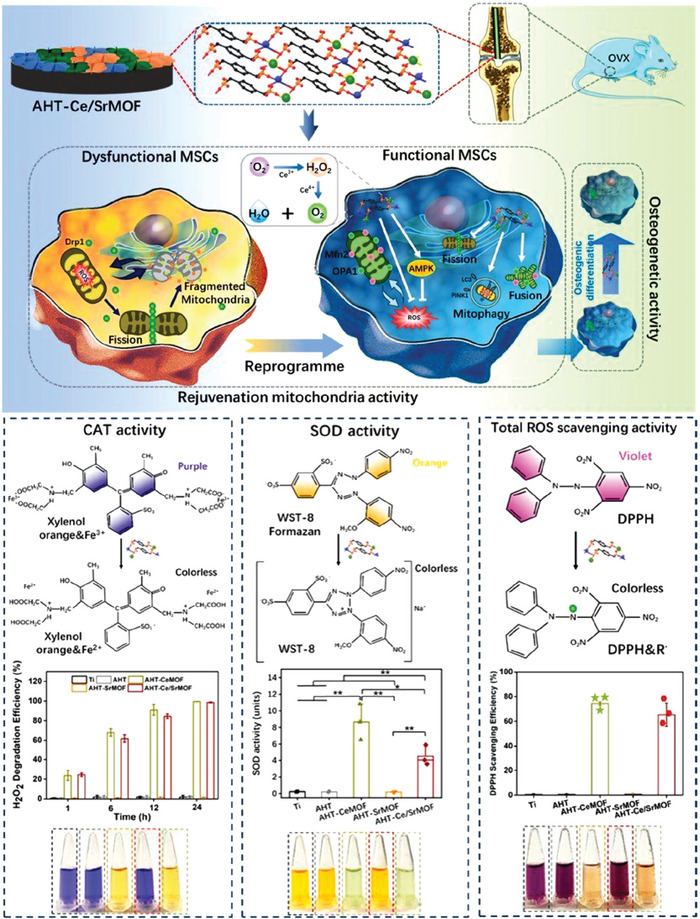
Schematic illustration of MOF‐coated Ti for improving osseointegration in the context of osteoporosis. The nanocatalytic activity of the MOF coating could scavenge excessive mitochondrial ROS in senescent MSCs to restore their osteogenic capacity. Reproduced with permission from Ref. [62]. Copyright 2022, American Chemical Society.

The pro‐bone healing effects of non‐calcium metal cations including Zn^2+^, Mg^2+^, Co^2+^, Sr^2+^, and Cr^2+^ are a hot topic in orthopedic research in recent decades. Specifically, these metal ions have positive roles in a variety of osteogenesis‐related events including bone formation and remodeling, owing to their biochemical similarity with Ca^2+^.^[^ ^61^ ^]^ Interestingly, these metal species could also be deployed as coordination centers for MOF synthesis, which would be gradually released from the MOF structures through spontaneous degradation. Consequently, employing MOFs containing these osteogenesis‐stimulatory metal ions for Ti implant coating could potentially enhance the eventual osseointegration efficiency through promoting peri‐implant new bone formation. For instance, Chen et al. synthesized biofunctional MOF structures through the coordination between Ce^4+^/Sr^2+^ ions and *p*‐xylylenebisphosphonate (PXBP) under hydrothermal conditions and used them for the coating of micro arc oxidized Ti implant surface, which could promote the osseointegration of the Ti implants even under osteoporotic conditions (**Figure** **6**).^[^ ^62^ ^]^ The bio‐MOF coating could be activated by the excessive ROS in the osteoporotic bone microenvironment in a self‐immolate manner, leading to the successful release of PXBP and Ce/Sr ions while ameliorating the overall ROS stress in the bone microenvironment. Here the Ce and Sr ions could be taken in by senescent MSCs and continuously decompose mitochondrial ROS into non‐toxic end products through redox cycling, leading to efficient mitochondrial normalization to restore their osteoblastic functions. Furthermore, the concurrently released PXBP could cooperatively inhibit osteoclast activity by suppressing osteoclastogenesis and inducing apoptosis of existing osteoclasts, thus rebalancing the bone formation and resorption in the osteoporotic bone microenvironment. These effects could act in a synergistic manner to improve osseointegration potential of Ti implants for treating osteoporotic fractures. Similarly, Chen et al. employed nanoscale ZIF‐8 MOF to modify Ti implant surface and found that it could release Zn^2+^ ions to stimulate the osteogenesis‐related genes in MG63 cells including ALP and RUNX2 and promote the mineralization of their ECM.^[^ ^63^ ^]^ Follow‐up study by the same group further elucidated that Zn^2+^ ions can stimulate ERK signaling in MSCs to achieve the pro‐osteogenesis effects. Yan et al. prepared Zr‐Fe‐based MOF structures through the coordination between Zr^4+^ ions, 1,1′‐dicarboxyferrocene and butyric acid and used them for Ti implant modification. The composite MOF coating could respond to endogenous H_2_O_2_ and exogenous NIR illumination in the context of osteosarcoma to drive Fenton reaction for eliminating the tumor cells, which would significantly enhance the surface hydrophilicity of the Ti implants to recruit MSCs.^[^ ^64^ ^]^ Meanwhile, the hydrophilic MOF surface would release Zr and Fe ions to boost the osteogenic potential of recruited MSCs to enhance local bone formation.

#### 3.2.2 MOF‐Enabled Antibacterial Activity for Ti Implant Stabilization

**Figure 7:**
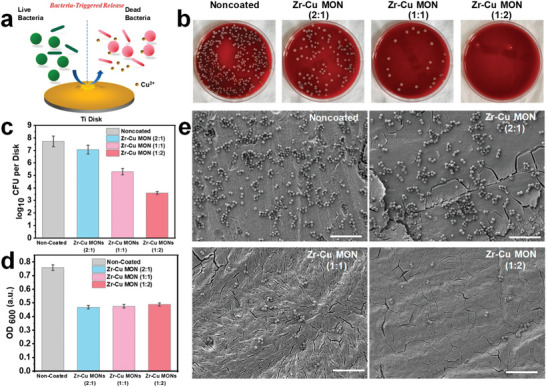
a) Mechanism of the MOF‐triggered antibacterial activities at bone‐implant interface. b) Impact of the Cu‐containing MOF coating on bacterial colony formation. c) Quantitative analysis of colony formation in panel b. d) MRSA proliferation rate after inoculation onto MOF surface with different Cu ion ratio. e) SEM images of MRSA growth on different MOF coatings. Reproduced with permission from Ref. [69]. Copyright 2020, American Chemical Society.

Despite the extensive sterilization of the Ti implants during surgery, implant infections still occur frequently and are acknowledged as a major cause of implant failure in the clinics.^[^ ^65^ ^]^ Generally, bacteria on the contaminated implants would trigger acute and chronical inflammation responses that eventually lead to the fibrous encapsulation of the implants, thus establishing an immunosuppressive niche for bacterial colonization and proliferation. Meanwhile, bacteria colonies adhered onto the Ti implant surface may further develop biofilms through secreting abundant extracellular matrix, leading to persistent infection.^[^ ^66^ ^]^ A major strategy to overcome the challenge of implant‐associated bacterial infection is to develop implants with intrinsic antibacterial activity, which should be able to eliminate the adhered bacterial and prevent biofilm formation. Remarkably, many of the metal species in common MOF structures also have considerable broad‐spectrum antibacterial activity, indicating their potential application for developing multifunctional Ti implants with dual antibacterial and pro‐osteogenesis capacities.^[^ ^67^ ^]^ Typically, Shen et al. synthesized a series of Mg/Zn hybrid MOFs (Mg/Zn‐MOF74) as functional Ti implant coating for dual antibacterial and pro‐osseointegration purposes. The Mg/Zn‐MOF74 coating could be gradually degraded after implantation to release the metal ions into the bone microenvironment as well as reducing its overall acidity.^[^ ^68^ ^]^ The resultant slightly alkaline peri‐implant microenvironment and Zn^2+^ ions could act in a cooperative manner to enable efficient elimination of adhered bacteria, thus abolishing post‐implantation bacterial infection. Furthermore, the concurrently released Zn^2+^ and Mg^2+^ ions could both stimulate the osteogenic activity of osteoblasts to promote new bone formation at the peri‐implant regions, eventually contributing to robust osseointegration of the Ti implants. Chu et al. synthesized para‐mercaptobenzoic acid (pMBA)‐capped gold nanoclusters (GNC) as amphiphilic anionic nanoscale ligand for the coordination‐enabled crosslinking with both tetravalent metal ions (Ti, Zr, Hf) and divalent metal ions (Cu, Zn) to form hybrid metalorganic framework by carefully controlling the Lewis acidity and coordination bonding geometry and used it for the coating of Ti implants (**Figure** **7**).^[^ ^69^ ^]^ The hybrid MOF coating retained the antibacterial activity of both p‐MBA‐capped GNCs and divalent metal ions by damaging bacterial membrane structures while demonstrating good chemical stability and non‐toxicity to human cells.

Several types of MOF structures have also demonstrated interesting light‐driven biophysical and biochemical activities due to the incorporation of photoreactive ligands and metal centers, offering novel opportunities for the localized elimination of peri‐implant bacteria through in situ photodynamic or photothermal therapy.^[^ ^70^ ^]^ Typically, by deploying photosensitizers as the organic ligands, MOFs could demonstrate considerable photochemical reactivity under light stimulation and generate abundant ROS, which could efficiently damage biomacromolecules in bacteria including lipids, proteins and genetic materials for evoking potent microbicidal effects. For instance, Li et al. synthesized porphyrin‐Cu MOF nanosheets (CuTCPP) and modified their surface with an atomic‐layer Fe_2_O_3_. The energy evolution pathways and charge transfer at the heterointerface allowed enhanced photodynamic activity under the excitation wavelength of 660 nm, which could synergize with the concurrently released Cu ions to efficiently inhibit bacterial growth.^[^ ^71^ ^]^ Alternatively, Yang et al. carbonized Zn^2+^‐rich ZIF‐8 MOF structures to prepare ZnO‐doped nanoparticles (ZnO‐CNPs) and coated their surface with a thermoresponsive gel layer (TRGL). The carbonized substrate has good photothermal conversion efficiency for NIR illumination at 808 nm. Under NIR treatment, the ZnO‐CNP‐mediated photothermal effect could induce the hydrophilic to hydrophobic conversion of the TRGL shell and enhance the affinity between ZnO‐CNP‐TRGL and the hydrophobic binding proteins on bacterial surface to form nanoparticle‐bacterial aggregates, after which the massive heat generation and Zn^2+^ release would synergistically eliminate the entrapped bacteria with a microbicidal efficiency of ≈ 100%.^[^ ^72^ ^]^ Overall, these studies collectively supported that designing multifunctional MOF coating on Ti implants for light‐controllable antibacterial treatment could be a promising strategy to prevent post‐implantation infection and improve implant stability, although several issues still need to be addressed from a translational perspective including photodynamic/photothermal stability of the MOF coatings, treatment repeatability and biocompatibility of photodegradation products.

### 3.3 MOF‐Coated Ti Implants as Drug Delivery Depots

**Figure 8:**
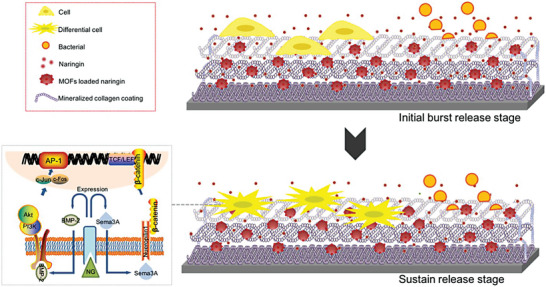
Mechanism of the MOF‐controlled naringin release from implant surface for improving osseointegration. Reproduced with permission from Ref. [74]. Copyright 2017, American Chemical Society.

In addition to their intrinsic biochemical reactivity, MOF structures have also been exploited for drug delivery applications owing to their large surface area, well‐defined porous structure and triggerable degradability. Consequently, MOF‐coated Ti implants could also be employed as therapeutic devices by incorporating cooperative or standalone treatment modalities.^[^ ^73^ ^]^ In a recent study by Yu et al., the authors first prepared MOF nanocrystals via coordination between Zr^4+^ and tetrakis(4‐carboxyphenyl) porphyrin through hydrothermal method, which were then loaded with naringin, a natural‐occurring antiosteoporosis drug that could stimulate the osteogenic function of osteoblasts, and further mixed with type I collagen to afford mineralized collagen coating on Ti surface via electrochemical deposition (**Figure** **8**).^[^ ^74^ ^]^ Compared with mineralized collagen coating loaded with free naringin, the MOF‐incorporated collagen‐coating successfully prevented burst‐like naringin release from the composite coating by physically trapping the naringin molecules with the MOF‐intrinsic porous structure, which significantly reduced the risk of associated toxicity while enabling long‐term pro‐osteogenesis effects due to sustained naringin release. Alternatively, Xiao et al. developed Mg‐Zn‐MOF74 implant coating for the localized delivery of dexamethasone (DEX), a clinically tested osteogenic drug. Owing to its well‐defined porous structures, the MgZn‐coating has large encapsulation capacity for DEX and potentiated sustained DEX release for stimulating peri‐implant osteogenesis, which could cooperate with the osteogenesis‐stimulatory functions and antibacterial activity of gradually released Mg^2+^ and Zn^2+^ ions to enable robust implant osseointegration.^[^ ^75^ ^]^ Taking advantage of the tunable pore widths, MOF‐based nanostructures have also demonstrated unique advantages for the delivery of macromolecular therapeutic substances such as peptide, aptamers, siRNAs and proteins, which may expand our arsenal for treating bone fractures.

## 4 Conclusion

In this review, we have comprehensively reviewed the recent advances in the development of MOF‐based coating for enhancing the osseointegration of Ti implants and discussed the relevant therapeutic mechanism. Current insights collectively suggest that the unique structural, physical, and chemical properties of MOF coatings provide practical solutions to address the clinical challenges associated with Ti implants including insufficient bone‐implant interaction at the implant interface, unsatisfactory osteogenic activity of peri‐implant osteoblasts and high risk of implant‐associated infections. Indeed, the tunable physical properties, versatile biochemical reactivity and potent drug delivery capacity of MOF coatings are highly favorable for the optimization of bioinert Ti implant surface toward better osteointegration efficacy in a clinical context.

Nevertheless, despite the promising prospects of MOF‐functionalization for Ti implant modulation, there are several important considerations and challenges in the development of biofunctional MOF coating for translational purposes. Notably, the most important prerequisite of biomedical systems for in vivo applications is biocompatibility. It is well‐established that metal species and organic ligands are the two major components of any MOF‐based materials, and the selection of metal species and ligands has fundamental impact on the biocompatibility profiles of the MOF coating. Typically, most MOF‐based biomaterials for in vivo applications choose essential elements as the coordination center such as Mg, Fe, Ca, etc., while avoiding toxic or non‐excretable elements such as Hg. It is also notable that the organic ligands in most of the current MOF designs have not been approved for clinical usage. Considering the fact that the coordinated metal species and organic ligands would be gradually decoupled in biological environment, the inevitable release of these organic ligands would induce significant health risks that cannot be overlooked both in the short term and long term. Therefore, most of the research interest in this area focuses on those less toxic MOFs including ZIF‐8, ZIF‐67, MIL‐53, MIL‐88, MIL‐100, UiO‐66, etc. Alternatively, there are also attempts to construct MOF‐based biomaterials using bioderived ligands including amino acids, peptides, nucleobases, carbohydrates and so on, which may improve the overall biocompatibility of the MOF coating after their eventual degradation in vivo. Furthermore, there is increasing interest to employ clinically approved small‐molecule drugs as the ligand struts for the complexation with bioactive metal centers, leading to a “minimalist” MOF design with self‐sufficient pro‐osteogenesis activity and total safety.

In addition, it is worth mentioning that the biochemical activity of the released metal ions is rather complex and may sometimes induce adverse toxic effects to impair implant osseointegration or cause local or systemic post‐implantation complications. Typically, it was recently demonstrated that Cu^2+^ and Ag^+^ evidently promoted the polarization of macrophages to M1 phenotype and stimulated the secretion of pro‐inflammatory cytokines, and it is thus anticipated that MOF coating incorporated with these elements would elevate the risk of local or systemic inflammation, leading to undesirable bone damage and autoimmune diseases. Moreover, the premature dissipation of the MOF coating due to spontaneous degradation would also impair its pro‐osseointegration efficiency and elevate the risk of implant loosening. Therefore, the selection of metal centers and biological stability of the MOF structures should be taken into consideration when designing MOF‐modified Ti implants for balanced pro‐osseointegration and safety performance.

The frequent micromotion and friction at bone‐implant interface also raise significant concerns regarding the stability of the MOF coating in the complex biomechanical environment, which may also profoundly affect their toxicity in vivo. Notably, mechanical wear and tear of the MOF coating would possibly compromise their structural integrity and release large amounts of MOF particles. It is worth mentioning that the variations in MOF size would not only substantially alter their cytotoxicity profiles but also trigger their redistribution at a systemic level, leading to unpredictable health risks. To improve the therapeutic performance and safety of MOF coating in the presence of various mechanical stresses, there are two major approaches including (1) enhancing the mechanical resilience of the MOF structures via chemical engineering and (2) modifying the MOF surface with mechanically flexible synthetic or bioderived macromolecules such as PEG and HA, although their potential efficacy in vivo remains to be investigated.

The increasing pace of population ageing around the world and the accompanied burden of various osteodegenerative diseases also raise new challenges for improving the osseointegration of Ti implants. Notably, clinical analysis collectively demonstrates that a series of common morbidities in middle‐aged or elderly people have substantial negative impact on the regeneration capacity and quality of the skeleton system and may critically impair the osseointegration of Ti‐based implants, of which the notable examples include osteoporosis, osteoarthritis, diabetes, etc. To overcome these disease‐associated challenges, there are emerging attempts to expand the therapeutic activity of MOF coating on Ti surface to develop drug‐device combination products with anti‐osteodegeneration capabilities. For instance, Shen et al. employed layer‐by‐layer self‐assembly technology to establish composite coating comprising Zn‐based MOFs and anti‐osteoporosis drug raloxifene (Ral) on Ti surface. Ral could be gradually released from the composite coating and reverse the osteoporosis symptoms through its estrogen‐agonistic functions to promote new bone formation and deposition, which could synergize with the anti‐osteoclastic function of release Zn^2+^ ions to enhance the osseointegration of Ti implants.^[^ ^76^ ^]^ Chen et al. constructed Mg‐Ga integrated nanosheet coating on Ti surface that could effectively scavenge protons in the osteoporotic bone microenvironment, which would thus promote the autophagic activity in recruited MSCs to enhance their osteoblastic differentiation while reducing osteoclast‐mediated bone resorption, eventually reversing the osteoporosis‐induced bone deterioration to achieve robust osseointegration.^[^ ^77^ ^]^ These MOF‐enabled drug‐device designs offer potential approaches for fracture management in patients with compromised bone regenerative capabilities.

## Conflict of Interest

The authors declare no conflict of interest.
